# Premature MicroRNA-1 Expression Causes Hypoplasia of the Cardiac Ventricular Conduction System

**DOI:** 10.3389/fphys.2019.00235

**Published:** 2019-03-18

**Authors:** Eva Samal, Melissa Evangelista, Giselle Galang, Deepak Srivastava, Yong Zhao, Vasanth Vedantham

**Affiliations:** ^1^Gladstone Institute of Cardiovascular Disease, San Francisco, CA, United States; ^2^Department of Medicine, University of California, San Francisco, San Francisco, CA, United States; ^3^Cardiovascular Research Institute, University of California, San Francisco, San Francisco, CA, United States; ^5^Department of Pediatrics, University of California, San Francisco, San Francisco, CA, United States; ^4^Biochemistry and Biophysics, University of California, San Francisco, San Francisco, CA, United States; ^6^Department of Genetics and Genomic Sciences, Mount Sinai Hospital, New York, NY, United States

**Keywords:** cardiac conduction system, cardiac development, microRNA, miR-1, Purkinje fibers, cardiac maturation, heart block

## Abstract

Mammalian cardiac Purkinje fibers (PFs) are specified from ventricular trabecular myocardium during mid-gestation and undergo limited proliferation before assuming their final form. MicroRNA-1 (miR-1), a negative regulator of proliferation, is normally expressed in the heart at low levels during the period of PF specification and outgrowth, but expression rises steeply after birth, when myocardial proliferation slows and postnatal cardiac maturation and growth commence. Here, we test whether premature up-regulation and overexpression of miR-1 during the period of PF morphogenesis influences PF development and function. Using a mouse model in which miR-1 is expressed under the control of the Myh6 promoter, we demonstrate that premature miR-1 expression leads to PF hypoplasia that persists into adulthood, and miR-1 TG mice exhibit delayed conduction through the ventricular myocardium beginning at neonatal stages. In addition, miR-1 transgenic embryos showed reduced proliferation within the trabecular myocardium and embryonic ventricular conduction system (VCS), a source of progenitor cells for the PF. This repression of proliferation may be mediated by direct translational inhibition by miR-1 of the cyclin dependent kinase Cdk6, a key regulator of embryonic myocardial proliferation. Our results suggest that altering the timing of miR-1 expression can regulate PF development, findings which have implications for our understanding of conduction system development and disease in humans.

## Introduction

The cardiac ventricular conduction system (VCS) is a multicomponent functional unit composed of specialized cardiomyocytes that transmit electrical impulses from the atrial to the ventricular myocardium. The proximal portion of VCS, the atrioventricular bundle, originates in the compact AV node, traverses the central fibrous body, and then bifurcates into the right and left bundle branches. Within each ventricle, the bundle branches ramify into conduction fascicles, which, in turn, arborize into a finely branched network of specialized cardiomyocytes known as Purkinje fibers (PF). At their distal ends, PFs directly activate working cardiomyocytes, leading to mechanically synchronous ventricular contraction. Malfunction and/or malformation of the Purkinje network can lead to conduction delay, dyssynchronous contraction, and tachyarrhythmias, and are important sources of morbidity in patients with congenital and acquired heart disease (Boyden et al., 2016).

PF myocytes are specified from a pool of bipotent ventricular cardiomyocyte precursors shortly after ventricular trabeculation and develop through a combination of slow proliferation and continued recruitment until late embryogenesis (Gourdie et al., 1995; Miquerol et al., 2010). The process of PF outgrowth continues into the perinatal period, after which the PF matures into its final physiological phenotype. While the regulatory determinants of this developmental program have not been fully established, a key factor in determining the functional properties of the whole unit is maintenance of the proper ratio of conduction system cells to working myocardium: PF hypoplasia leads to conduction defects (Jay et al., 2004; Kim et al., 2016) while PF excess causes reduced contractile force and arrhythmias (Park et al., 2013). In the murine heart, PFs comprise only about 1% of ventricular myocytes, partly because the rates of proliferation of PF cells and their trabecular myocardial precursors are substantially lower than that of working myocytes in the compact myocardial layer (Miquerol et al., 2010).

Among the key regulators of ventricular myocardial proliferation in the late developmental and perinatal period is the muscle-specific microRNA, miR-1. During mammalian cardiac development, miR-1 expression is detectable in cardiomyocytes shortly after cardiogenesis and is steeply upregulated in the postnatal period, when miR-1 expression comprises a large percentage of the total microRNA in the heart. MiR-1 is expressed throughout atrial and ventricular myocardium, and within the specialized conduction tissue, but not in non-myocyte cells within the heart (Rao et al., 2009; Cao et al., 2012). Overexpression of miR-1 under the control of the Myh7 promoter results in early embryonic lethality, partly due to decreased ventricular cardiomyocyte proliferation. Conversely, homozygous deletion of one miR-1 allele (miR-1-2) leads to increased proliferation in postnatal hearts as compared to WT hearts, suggesting that miR-1 is involved in negatively regulating proliferation during perinatal cardiac maturation (Zhao et al., 2005, 2007).

In addition to the effects on proliferation, gain of function models have demonstrated that excess miR-1 can lead to arrhythmias including atrioventricular block and ventricular tachycardia in adult animals. These arrhythmias have been attributed to the capacity of miR-1 to inhibit expression of various gap junctions, ion channels, calcium handling proteins, and other electrophysiologically salient targets (Yang et al., 2007; Terentyev et al., 2009; Zhang et al., 2013; Su et al., 2017). In addition, previous work has demonstrated that chronic exposure to elevated miR-1 levels leads to abnormal cardiac growth and contractile dysfunction, with inhibition of sarcomere assembly (Ai et al., 2012).

Here, we extend these studies by focusing on PF development and maturation using a transgenic mouse in which the Myh6 promoter drives miR-1 overexpression in the heart. Several studies have demonstrated that Myh6 promoter activity is present during embryogenesis beginning around embryonic day (E) 10, albeit at lower levels than in the adult heart (Lyons et al., 1990; Subramaniam et al., 1991; Toib et al., 2012). Consistent with these previous results, we detected up-regulation of miR-1 in our Myh6-miR-1 transgenic embryos during the critical period of VCS specification and outgrowth (between E10.5 and E14.5). We further show that Myh6-miR-1 animals have a hypoplastic VCS, reduced proliferation of PF precursors, and early-onset electrocardiographic abnormalities including broadened QRS complex (reflecting slowed transit through the VCS). Finally, we defined the cyclin-dependent kinase Cdk6 as a target of miR-1 in the mouse heart and a possible contributor to the observed hypoplasia. Our results demonstrate the importance of the timing of miR-1 expression relative to cardiac development and conduction system morphogenesis.

## Materials and Methods

### Use of Genetically Modified Mice

UCSF is accredited by Association for Assessment and Accreditation of Laboratory Animal Care International (AAALAC), effective June 14, 2004, and experimentation was carried out in strict accordance with AAALAC guidelines. All animal experiments were reviewed and approved by the Institutional Animal Care and Use Committee (IACUC) at UCSF.

Generation of Myh6-miR-1 TG (hereafter: miR-1 TG) has been reported previously (Zhao et al., 2005). CCS-LacZ mice were originally generated by Glenn Fishman’s lab (Rentschler et al., 2001) and were provided to us by Benoit Bruneau. Irx3-LacZ mice were generated by Chi-Chung Hui’s lab and provided by Benoit Bruneau (Zhang et al., 2011).

### Histology

Embryonic, neonatal and adult miR1-TG and WT littermates were sacrificed and hearts were removed and processed for hematoxylin-eosin staining using standard techniques. For whole-mount Bluo-Gal staining, adult hearts were bisected, fixed overnight in formalin, and incubated with a solution containing 1 mg/mL halogenated indolyl-β-galactoside (Bluo-Gal, Invitrogen, Catalog No. 15519028) for 24 h at room temperature, followed with washing and fixation. To facilitate comparison between genotypes, WT and TG littermate hearts were processed and stained in parallel using the same staining solution for identical durations. Hearts were photographed under identical lighting conditions after clearing in 2:1 benzyl alcohol-benzyl benzoate (Sigma-Aldrich, Catalog No. 305197 and B6630, respectively).

### Morphometric Quantification of His Purkinje System

For quantification of whole-mount Irx3-LacZ staining, we used 5 P5 miR-1 TG hearts and 3 WT littermates that were each heterozygous for Irx3-LacZ. All hearts were fixed and stained with Bluo-Gal in parallel, then cleared for whole-mount photography under identical lighting conditions. Images of PFs were desaturated and color inverted to make bluo-gal positive areas have higher pixel intensity. Pixel intensity was then integrated across each image, averaged, and normalized to the average WT value to arrive at relative PF density. These values were averaged and an unpaired *t*-test was used to test for difference from the WT with *p* < 0.05 deemed significant.

### Whole-Mount *in situ* Hybridization

RNA probe for Bmp10 was generated by *in vitro* transcription in the antisense direction using Ambion Message Machine kit (AM1340), followed by labeling with the DIG RNA labeling kit (Roche, Catalog No. 11277073910). 3 miR-1 TG and 3 WT E10.5 embryos were dissected and processed for *in situ* hybridization as previously described (Vedantham et al., 2013).

### Immunohistochemistry

For immunohistochemistry and acetylcholinesterase staining, hearts were fixed overnight in formalin, washed in PBS, and moved through a sucrose gradient into 30% sucrose overnight. They were then embedded in Optimal Cutting Temperature Compound (Fisher Scientific, Catalog No. 23-730-571) prior to cryosectioning. Sections were washed in PBS, blocked in 5% goat serum for 1 h, incubated with primary antibody (Phosphohistone H3 – 1:500, Millipore Sigma 06-570, Hcn4 – 1:200, Alomone Labs #APC-052; Connexin-40 – 1:200, Alpha Diagnostics Cx40-A; Connexin-43 – 1:100, Sigma SAB 4501173; NaV1.5 – 1: 200, Alomone Labs #ASC-005; Beta-Galactosidase – 1:200, Abcam Ab9361), washed, and incubated for 1 h with a secondary antibody (Alexa Fluor, Life Technologies) before a final wash and mounting in Vectashield with DAPI (Vector Laboratories, Catalog No. H-1200). To assess mitotic index, the number of phosphohistone H3-positive cells within the trabecular myocardium and compact myocardium of each section was determined, and then divided by the total number of nuclei. For determination of mitotic index specifically in VCS, beta-galactosidase+ myocardium in sections from Irx3-LacZ;miR-1 TG and Irx3-LacZ littermates was manually segmented using ImageJ, and phosphohistone H3-positive cells within this compartment were counted and divided by the total number of nuclei in the beta-galactosidase positive cells. A total of 3 hearts for each genotype was used with at least 2 distinct sections examined per heart. Statistical comparison between WT and TG was performed with unpaired *t*-tests with *p* < 0.05 deemed significant.

### Quantification of Cardiomyocyte Size and Number

To measure cardiomyocyte cross sectional area, photomicrographs taken from wheat-germ-agglutinin stained sections (4 sections per heart from 3 WT and 3 miR-1 TG hearts) were loaded onto ImageJ and 10 cardiomyocytes sectioned *en face* were selected at random from each section (total of 120 per genotype) and manually segmented for area measurements. Cross sectional areas between the two genotypes were compared using two-tailed *t*-tests. To estimate cellularity, a tangent line to the epicardium was drawn and then a second line perpendicular to the tangent was drawn between the endocardium and epicardium. Cell diameters were counted along that line. This process was repeated in the same sections from 3 positions in the LV and at 3 sites in the RV, each with a distinct tangent line along the epicardium. Statistical comparisons were made with unpaired *t*-tests and *p* < 0.05 was deemed statistically significant.

### Optical Projection Tomography

After whole-mount Bluo-Gal staining, neonatal hearts were embedded in low-melt agarose, cleared with 2:1 benzyl alcohol:benzyl benzoate, and imaged with a Bioptonics optical projection tomography scanner model 3001M (MRC Technology, Edinburgh, UK). Image processing and 3D reconstruction was performed with NR Recon (Bioptonics) and videos were generated with Amira (Visage Imaging).

### Quantitative Analysis of Gene Expression

The ventricles of hearts from neonatal and adult mice were dissected in nuclease-free PBS and homogenized in Tri-Reagent with a bead homogenizer system (Bullet Blender, Next Advance, model BBX24). Total RNA was prepared from the aqueous fraction with isopropanol-ethanol precipitations. MicroRNA quantitative PCR and messenger RNA quantitative PCR were performed on an Applied Biosystems 7900HT machine with Taqman reagents (Life Technologies). PCR primers used are given in Supplementary Table S1.

For quantification of connexin-40 plaque intensity, individual connexin-40 plaques were manually segmented and fluorescence intensity was measured with image J with subtraction of background fluorescence from a manually selected connexin-40 negative adjacent region. At least 5 sections were examined per heart for 3 hearts of each genotype and statistical comparisons were performed with a Mann–Whitney test with *p* < 0.05 deemed significant.

### Western Blot

Adult WT and miR-1 TG mouse hearts were snap-frozen and ground to a fine powder using a mortar and pestle in the presence of liquid nitrogen. Powdered hearts were lysed in RIPA buffer using a homogenizer, and Bradford assays were performed on the cleared lysates. Fifty micrograms of total protein was run on a 10% tris-glycine gel, and transferred in 20% methanol onto a PVDF membrane (100V for 1.5 h at 4°C). The blot was blocked in 5% milk in TBST (0.05% tween-20) before incubation with anti-Cdk6 antibody (sc-7181, 1:1000 dilution) for 16 h at 4°C. HRP-conjugated secondary antibody (1:5000 for 1 h) was then applied and the blot was developed using Pierce ECL substrate (Thermo Fisher Scientific, Catalog No. 32106). For endogenous control, blots were rinsed, blocked, and re-probed with GAPDH antibody (SC-2313, 1:2500 dilution) following the same protocol as for Cdk6 detection.

### Cell Culture and Transfections

For knockdown studies, HL-1 cells were maintained in Claycomb media supplemented with 10% fetal bovine serum, 100 units/mL streptomycin/penicillin, 2 mM gutamine, and 0.1 mM norepinephrine. Confluent HL-1 cells were transfected with miR-1 inhibitor (Dharmacon, Catalog No. IH-310376-08-0002) by Amaxa nucleofection and harvested 48 h later for gene expression analysis as described above. All assays were performed in triplicate and data is expressed as mean ± standard deviation. Unpaired *t*-test was used to calculate statistical significance with *p* < 0.05 deemed significant.

### Adult and Neonatal ECG Recording

For adults, mice were administered 2% isoflurane and placed on a warmed platform with continuous temperature monitoring. Subcutaneous needle electrodes (AD Instruments, MLA1203A) were placed in limb lead configurations and signals were acquired using the PowerLab 4/30 analog-to-digital converter (ML866P) and a Dual Bioamp signal conditioner (AD Instruments, ML135), with offline data analysis using ChartPro software (AD Instruments, ML866P). Neonatal ECGs were performed as above without isoflurane and continuous temperature monitoring. Following Boukens et al. (2013), QRS intervals were measured in limb lead aVF configuration in which the measured QRS end is contemporaneous with the end of ventricular activation. Data are displayed as mean ± standard deviation, and statistical comparison was performed using unpaired *t*-tests with *p* less than 0.05 deemed significant.

### Mouse Transmitter Implantation and Recording of Awake ECGs

For transmitter implantation, adult miR-1 TG and WT mice were first anesthetized with 2% isoflurane, after which the thorax was epilated and sterilized with betadine. A 2 cm skin incision was made lateral to the spine and a subcutaneous pocket was created to hold the transmitter (ETA-F10, Data Sciences International, St. Paul, MN, United States). Sterilized recording leads (Data Sciences International) were tunneled to the left lower thorax and to the anterior right shoulder through small incisions and were sutured to intercostal and pectoralis muscles, respectively. The generator was anchored to the dorsal thoracic musculature using a suture, and the leads and generator were placed in the subcutaneous pocket, after which the incision was closed. Animals were allowed to recover for 8 days prior to initiating recording. Recovered miR-1 TG and WT adult mice with transmitters were housed in individual shielded cages and continuous ECG tracings were recorded for 24 h using the DSI telemetry system. ECGs were analyzed for heart rate, intervals, and the presence of arrhythmias offline using DSI software. Data for intervals are displayed as mean ± standard deviation, and statistical comparison was performed using unpaired *t*-tests with *p* less than 0.05 deemed significant.

### Mouse Echocardiography

Cardiac dimensions and function were assessed by measuring left ventricular dimensions with M-Mode in gently restrained unanesthetized 8 week old mice using a Vevo770 ultrasound machine (Visual Sonics). Statistical comparison was performed using unpaired *t*-tests with *p* less than 0.05 deemed significant.

### Measurement of Heart Weight to Body Weight Ratio

Mice at neonatal stage P0 (*n* = 5 for WT and TG), 2 weeks (*n* = 3 for WT; *n* = 6 for TG), 6 weeks (*n* = 5 for WT and TG), and 17 weeks of age (*n* = 4 for WT and TG) were weighed prior to euthanasia and removal of hearts. Residual blood was removed from hearts with gentle pressure and hearts were dissected free of extra-cardiac tissue. Hearts were washed in PBS, patted dry, and weighed. Statistical comparisons were made with unpaired *t*-tests and *p* < 0.05 was deemed statistically significant.

## Results

### Timing of MiR-1 Overexpression in MiR-1 TG Hearts

**FIGURE 1:**
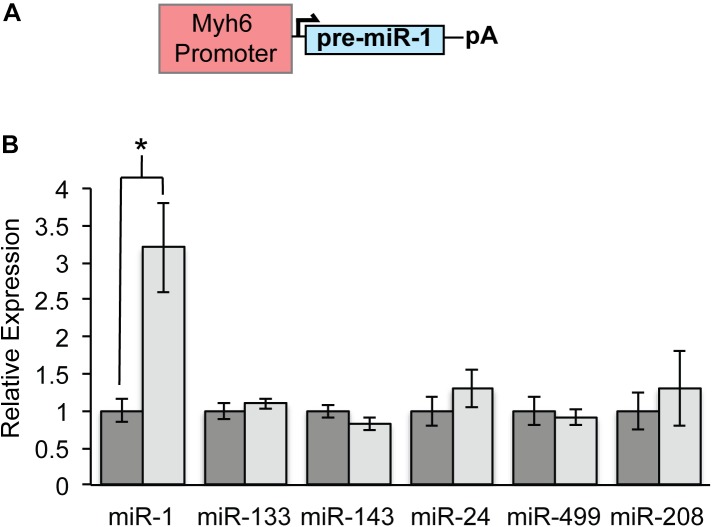
Specific Overexpression of MicroRNA-1 in Myh6-MiR-1-TG Mice. **(A)** The Myh6 promoter was cloned upstream of pre-miR-1-2 for pronuclear injection. **(B)** Quantitative PCR demonstrated that 8 weeks old miR-1 TG mice had about threefold overexpression of miR-1 as compared to WT littermates. Expression of other cardiac-enriched microRNAs was unchanged. “^∗^” denotes *p* < 0.05.

Myh6-miR-1 transgenic mice (hereafter, miR-1 TG) were generated for an earlier study using pronuclear injection of a construct bearing the Myh6 promoter upstream of pre-miR-1 and were previously reported (Figure 1A; Zhao et al., 2005). Quantitative PCR for mature miR-1 in ventricular myocardium from 8 week old mice showed threefold up-regulation of miR-1 as compared to WT littermates, while other cardiac microRNA levels were unchanged (Figure 1B).

**FIGURE 2:**
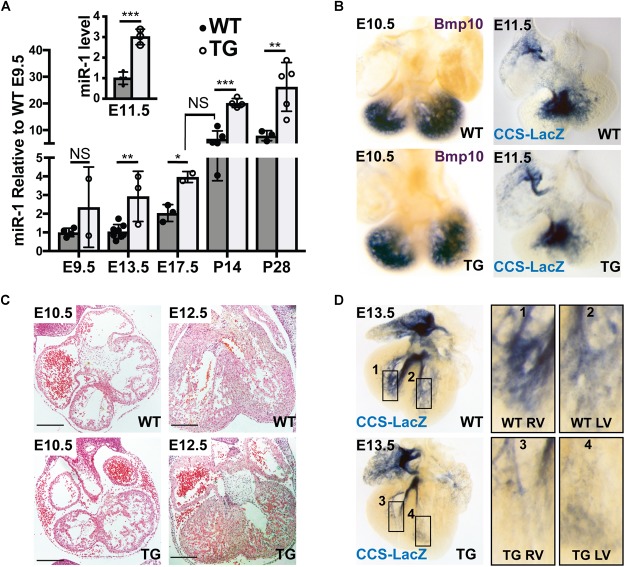
Hypoplasia of Ventricular Conduction System in miR-1 TG Embryos. **(A)** Quantitative PCR for mature miR-1 transcript in miR-1 TG hearts versus WT hearts at several stages of development shows that miR-1 expression is elevated in mid-gestation embryos and rises to adult levels in the perinatal period, anticipating the normal developmental upregulation of miR-1. The inset shows relative expression of miR-1 versus WT at E11.5. “^∗^” denotes *p* < 0.05, “^∗∗^” denotes *p* < 0.01, and “^∗∗∗^” denotes *p* < 0.001. **(B)** Whole-mount *in situ* hybridization at E10.5 for *Bmp10* in WT (top, left) and TG (bottom, left) and VCS development at E11.5 as assessed by whole-mount bluo-gal staining of a miR-1 TG heart (bottom, right) alongside a WT littermate (top, right) in the CCS-LacZ background. Note the similar distribution of reporter expression. **(C)** Light microscopic examination of embryonic hearts sectioned at E10.5 (left) and E12.5 (right) in WT (top) and miR-TG (bottom). Scale bars = 200 microns. **(D)** Whole-mount imaging of CCS-Lacz reporter activity at E13.5, after the onset of miR-1 upregulation in miR-1 TG mice, demonstrates a striking paucity of VCS tissue in the transgenic heart (bottom row) versus a WT littermate (top row). Panels 1–4 on the right show magnified images of developing RV (1,3) and LV (2,4) PF density in WT (1,2), and TG (3,4) hearts.

Because previous work has established that the Myh6 promoter is active during embryogenesis, we tested for up-regulation of miR-1 expression at several embryonic time points using quantitative PCR on RNA isolated from WT and miR-1 TG littermates. These data established the onset of miR-1 overexpression in miR-1 TG animals between embryonic day (E) 9.5 and E13.5, a critical window within which PF specification and outgrowth occurs (Figure 2A). Notably, while miR-1 levels in miR-1 TG embryonic hearts rose rapidly during embryogenesis, they did not approach WT adult levels until relatively late in embryogenesis. Embryonic hearts of miR-1 TG and WT littermates were examined histologically before the onset of miR-1 expression and no gross defects in cardiac morphogenesis were observed. MiR-1 TG animals were born at expected frequency with no evidence of embryonic lethality.

### Embryonic Malformation of Ventricular Conduction System in miR-1 TG Mice

To test whether exposure of the developing heart to elevated miR-1 levels affected VCS development, we began by examining the VCS and its precursors before E11.5, when miR-1 levels begin to rise in the miR-1 TG embryos but before they reach WT adult levels. *In situ* hybridization for Bmp10, a molecular marker for trabecular myocardium, revealed no difference in ventricular expression pattern between miR-1 TG and WT at E10.5, suggesting that the specification of VCS precursors was grossly unaltered in miR-1 TG animals (Figure 2B, left panels). We then crossed the miR-1 TG animals to the conduction system reporter mouse line, CCS-LacZ and examined embryos at different time points. As assessed by CCS-LacZ reporter activity, WT and TG hearts also had a similar appearance of the developing VCS at E11.5 (Figure 2B, right panel), confirming that VCS development is not perturbed prior to the onset of miR-1 overexpression in the miR-1 TG animals.

Histological comparison of E10.5 with E12.5 hearts, however, revealed that while trabeculations were well formed in the miR-1 TG hearts, ventricular myocardial growth (including trabecular growth) was diminished in miR-1 TG hearts after E11.5, when miR-1 levels rise in the miR-1 TG animals (Figure 2C). Similarly, beginning at E13.5, when miR-1 levels in miR-1 TG embryos approach WT adult levels, we observed a striking reduction in VCS tissue in CCS-LacZ transgenic embryos (Figure 2D). To confirm that the reduction in ventricular growth and VCS development at E12.5 and E13.5 in miR-1 TG embryos could be attributed to elevated miR-1 expression, we assessed miR-1 levels at E11.5 in miR-1 TG and WT animals and observed a threefold increase in miR-1 expression by this developmental stage (Figure 2A, inset).

### Ventricular Conduction System Structure and Function in MiR-1 TG Neonates

**FIGURE 3:**
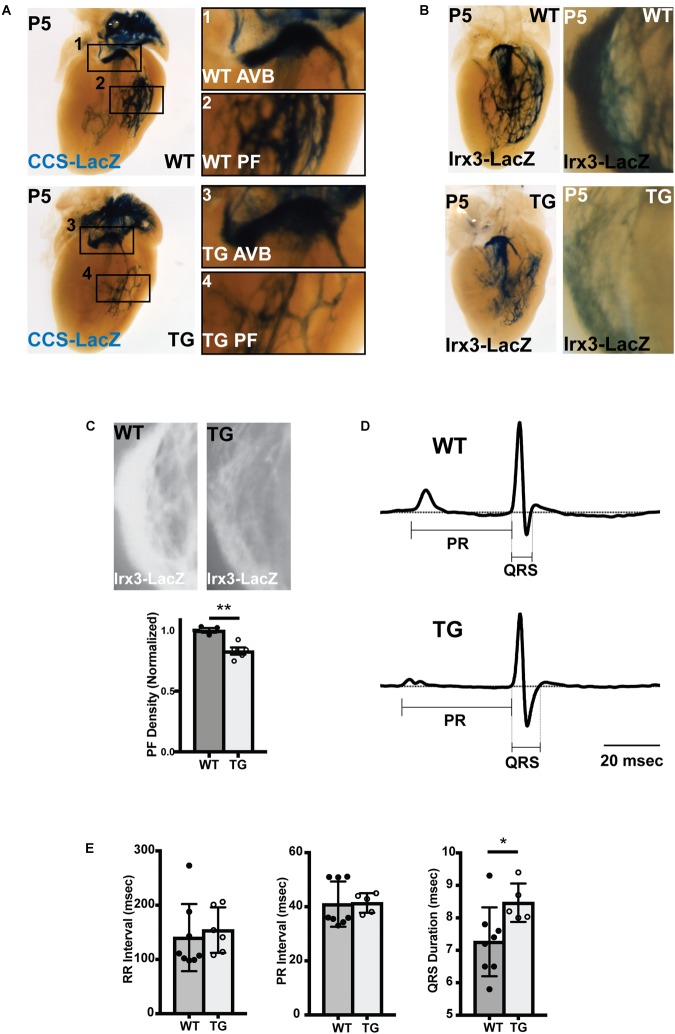
Abnormal Conduction System Structure and Function in MiR-1 TG Neonatal Mice. **(A)** Whole mount imaging of CCS-LacZ reporter activity in P5 hearts shows a reduction in PF density in the TG heart (bottom, left) versus the WT littermate (top, left). The magnified panels 1–4 (right) demonstrate atrioventricular bundle tissue (AVB; 1,3) and Purkinje Fiber (PF) density (PF; 2,4) in the WT (1,2) and miR-1 TG hearts (3,4). **(B)** Whole-mount imaging of reporter activity in WT (top) and TG (bottom) P5 hearts in the Irx3-LacZ background. Left panels show imaging of the left bundle branch and left-sided Purkinje fibers. Right panels show magnified images of right ventricular PFs. **(C)** Density of PFs in WT (*n* = 3) and miR-1 TG (*n* = 5) hearts were quantified from images of reporter activity in Irx3-LacZ hearts. Representative images for quantification are shown at top. The density of PFs was significantly reduced in miR-1 TG hearts, as shown in the bottom graph (“^∗∗^” denotes *p* < 0.01). **(D)** Averaged lead aVF ECG tracings recorded from a neonatal WT mouse (top) and a miR-TG littermate show prolongation of the QRS duration in the miR-1 TG, reflecting slowed conduction through the VCS. **(E)** RR, PR, and QRS intervals quantified from WT and miR-1 TG mice (mice obtained from 3 distinct litters, *n* = 8 for WT and *n* = 5 for TG, “^∗^” denotes *p* < 0.05).

To test whether the decrease in PF density persisted into the neonatal period, miR-1 TG;CCS-LacZ hearts were examined at P5. Consistent with the embryonic findings, we observed a clear reduction in PFs in the miR-1 TG mice (Figure 3A). Of note, we observed no reduction in CCS-LacZ reporter activity in the AV bundle, excluding the possibility that global reduction in CCS-LacZ promoter activity is responsible for the PF abnormalities that we observed. We also assessed the VCS by crossing miR-1 TG animals into the Irx3-LacZ reporter line, in which reporter activity is restricted to the VCS. We observed a striking reduction in PFs in the miR-1 TG animals in this line as well (Supplementary Video S1). Quantification of PF density by measurement of Irx3-LacZ reporter staining intensity, as shown in Figure 3B,C, demonstrated a robust and statistically significant reduction in 5 miR-1 TG hearts as compared to 3 WT littermate hearts. Taken together, these data demonstrate that premature up-regulation of miR-1 expression during embryogenesis causes PF hypoplasia.

We also assessed expression of critical functional genes in postnatal (P10) VCS cells, including Connexin-40 and Nav1.5, which contribute to the rapid conduction phenotype of these cells. While we did not observe any difference in Nav1.5 expression throughout the subendocardial myocardium, quantitative immunohistochemistry of VCS Connexin-40 expression revealed a 45% reduction in the intensity of connexin-40 plaques in miR-1 TG VCS cells as compared to WT littermates, demonstrating an effect of miR-1 on gene expression in VCS cells in addition to an effect on cellularity (Supplementary Figures S1A,B). Consistent with previous work on miR-1, we also observed moderate reduction in Connexin-43 staining in the ventricular working myocardium of miR-1 TG hearts (Supplementary Figure S1C).

To test whether the VCS hypoplasia and reduced Cx-40 and Cx-43 expression were associated with VCS dysfunction, we performed electrocardiograms in neonatal mice derived from 3 separate litters (*n* = 5 for miR-1 TG and *n* = 8 for WT). We measured the RR interval (reflecting heart rate), the PR interval (reflecting conduction through the AV node) and the QRS interval (reflecting conduction along the VCS). We found that while RR and PR intervals were not changed between miR-1 TG neonates and their littermates, the QRS interval was significantly increased in the miR-1 TG mice (*p* < 0.05), demonstrating isolated dysfunction of the VCS in hearts with PF hypoplasia and dysregulation of gene expression (Figure 3D,E).

### Conduction System Hypoplasia and Dysfunction Persists in Adult MiR-1 TG Hearts

**FIGURE 4:**
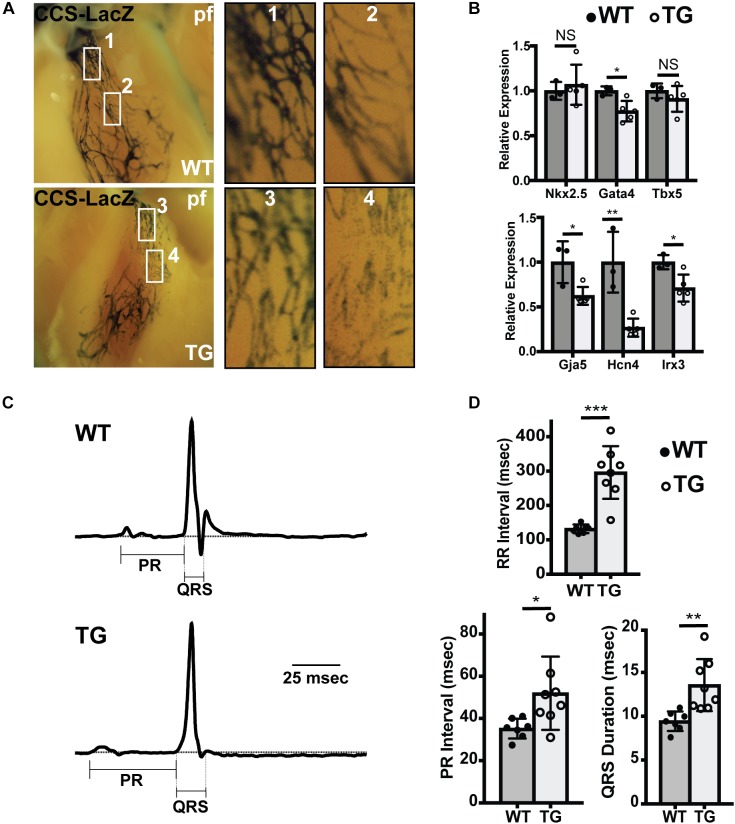
Abnormal Conduction System Structure in Adult MiR-1 TG Mice. **(A)** Whole mount imaging after fixation and bluo-gal staining of the ventricular conduction system in WT (top) and miR-1 TG (bottom) hearts in the CCS-LacZ background. PF density is reduced in the miR-1 TG adult hearts. **(B)** Quantitative PCR for genes enriched in PFs demonstrates decreased expression of conduction system genes and either unchanged or only modest changes in core cardiac transcription factors. “^∗^” denotes *p* < 0.05, “^∗∗^” denotes *p* < 0.01, and “^∗∗∗^” denotes *p* < 0.001; NS, non-significant. **(C)** Averaged ECG tracings derived from limb lead aVF from 8 weeks old WT (top) and miR-1 TG mice (bottom) demonstrated prolonged PR and QRS in the miR1-TG. **(D)** Comparison of electrocardiographic intervals in adult WT mice (*n* = 7) and miR-1 TG littermates (*n* = 8) demonstrate prolonged RR, PR, and QRS intervals in the miR-1 TG mice, reflecting widespread conduction system dysfunction.

To test whether conduction system hypoplasia persisted in adult hearts, we crossed miR-1 TG mice with the CCS-LacZ line, in which the entire VCS is labeled with LacZ, and performed whole-mount bluo-gal staining in adult mice at 8 weeks. Similar to findings in neonates and embryos, we observed reduction in the caliber and density of PFs in miR-1 TG mice as compared to WT littermates (Figure 4A).

If the ratio of PF to working myocardium is reduced in miR-1 TG animals, we reasoned that there should be relatively lower expression of PF enriched genes in the ventricles of TG hearts. To test this directly, we performed quantitative PCR to assess gene expression in ventricular tissue dissected from miR-1 TG and WT hearts. Whereas expression of pan-ventricular cardiac transcription factors Nkx2.5 and Tbx5 were not changed between the two genotypes, and Gata4 expression was only mildly reduced, expression levels of genes highly enriched in PF, including Gja5, Hcn4, and Irx3, were reduced by 38, 73, and 29 percent, respectively (Figure 4B).

Finally, to test for functional abnormalities of the VCS in adult miR-1 TG mice, we performed electrocardiograms in anesthetized adult miR-1 TG mice. Compared to their WT littermates, miR-1 TG mice exhibited slower heart rate, prolonged PR interval (reflecting delayed conduction through the AV node) and prolonged QRS duration, reflecting delayed conduction through the VCS (Figure 4C,D).

While prolonged QRS duration is likely a consequence of the VCS abnormalities we defined (hypoplasia and reduced connexin-40 expression), the PR interval increase is a distinct electrophysiological abnormality that suggests abnormal function of the AV node and/or AV bundle. We used acetylcholinesterase staining, which marks central conduction tissue, and observed no gross difference between miR-1 TG animals and WT animals in the size of the AV node or AV bundle. We further assessed these structures with immunohistochemistry for Hcn4 (AV node) and connexin-40 (AV bundle), and observed no differences in morphology of these structures between WT and miR-1 TG hearts although staining intensity for these proteins was reduced in miR-1 TG hearts (Supplementary Figures S1D,E). Thus, slowed conduction through the structurally intact AV node is likely due to functional abnormalities caused by chronic exposure to elevated miR-1 levels, as has been described previously, and not to hypoplasia (Zhang et al., 2013).

### MiR-1 Inhibits Proliferation of VCS Precursors

**FIGURE 5:**
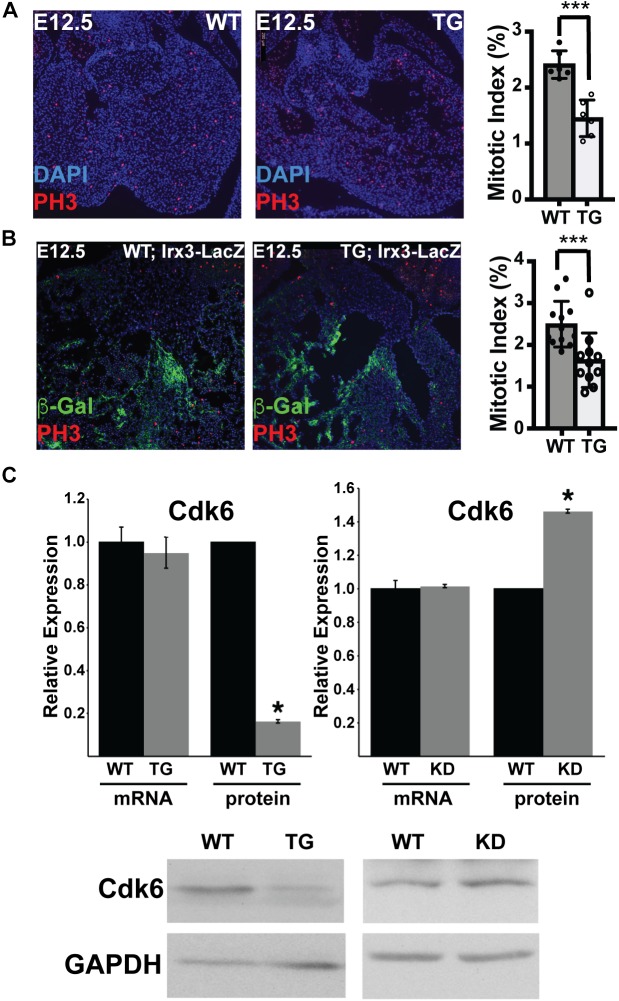
MiR-1 Negatively Regulates VCS Proliferation and Cdk-6 Protein Expression. **(A)** Phosphohistone H3 staining in WT and miR-1 TG hearts at E12.5 shows a reduction in PH3+ cells in transgenic animals as compared to WT. The graph shows the percentage of trabecular nuclei that are PH3+ in each genotype. “^∗∗∗^” denotes *p* < 0.001. **(B)** Co-staining of phosphohistone H3 and beta-galactosidase in Irx3-LacZ and Irx3-LacZ; MiR-1 TG E12.5 hearts demonstrates reduced mitotic index specifically in the developing VCS. The graph shows the percentage of beta-galactosidease nuclei that are also phosphohistone H3 positive “^∗∗∗^” denotes *p* < 0.001. **(C)** mRNA level of Cdk6 is unchanged in postnatal hearts from miR-1 TG versus WT littermates, while protein level is significantly decreased, consistent with translational regulation. Knockdown of miR-1 in HL-1 cells results in upregulation of Cdk6 at the protein level. ^∗^ denotes *p* < 0.05.

VCS cells are specified and differentiate from bipotent proliferative trabecular myocardium. Therefore, VCS hypoplasia could have resulted from impaired trabeculation, impaired specification of the VCS, or from reduced proliferation of specified VCS and/or trabecular precursor cells. Because miR-1 negatively regulates proliferation in other contexts, we hypothesized that increasing miR-1 in embryonic hearts might have led to reduced proliferation within the trabecular myocardial compartment, which, in turn, would have reduced the available pool from which VCS cells are ultimately recruited. To test this hypothesis, we used phosphohistone H3 immunohistochemistry on E12.5 embryonic heart sections from miR-1 TG and WT littermates (3 animals per genotype from 2 different litters with 2 sections per embryo, Figure 5A). We manually segmented the trabecular myocardium and in this compartment we found a significantly reduced mitotic index in the TG animals as compared to WT littermates, confirming our hypothesis of hypoproliferation of VCS precursors. We also segmented and assessed mitotic index in the compact myocardium and observed a similar reduction in the miR-1 TG animals (4.2% in WT versus 2.9% in miR-1 TG, *p* < 0.05).

Finally, to test directly whether proliferation is reduced in the VCS compartment, we stained sections from E12.5 Irx3-LacZ;MiR-1TG and Irx3-LacZ littermate embryos for beta-galactosidase expression and phosphohistone H3 (3 animals per genotype from 2 different litters with 2 sections per embryo). Mitotic index specifically within the beta-galactosidase + (Irx3 + VCS) cells showed a significant reduction in miR-1 TG as compared to WT cells, confirming that early miR-1 up-regulation results in decreased proliferation of VCS precursors (2.5% in WT versus 1.6% in miR-1 TG, *p* < 0.001, Figure 5B).

### MiR-1 Regulates Cdk6 Expression

Cdk6, an established miR-1 target in rat models, acts upstream of the pocket proteins, regulators of trabecular proliferation and conduction system cellularity (Liao et al., 2016). We therefore tested whether Cdk6 might also be a target of miR-1 in the mouse heart, and thereby explain the proliferation defect observed in trabecular myocardium. Cdk6 mRNA levels were not different between TG and WT littermates (Figure 5C). Cdk6 protein levels, however, were markedly reduced in miR-1 transgenic hearts, consistent with posttranscriptional down-regulation by miR-1. Furthermore, knockdown of miR-1 in cultured HL-1 cells increased Cdk6 protein levels (Figure 5C). Taken together, these results suggest that a miR-1/Cdk6 pathway may also be relevant to miR-1’s function i*n vivo* during the postnatal period when cardiomyocyte proliferation slows.

### Abnormal Cardiac Growth in Adult MiR-1 TG Hearts

Consistent with previous work, additional studies performed on adult miR-1 TG adult mice revealed age-dependent ventricular enlargement, arrhythmias including AV block, and premature death as compared to WT controls (Supplementary Figure S2; Ai et al., 2012; Zhang et al., 2013). Notably, despite the conduction abnormalities, ventricular function as assessed by echocardiography was preserved at 8 weeks, although we did observe ventricular chamber enlargement. This finding, along with preserved wall thickness and increased cardiac mass, suggested the presence of cardiac hypertrophy (Supplementary Table S2). To test directly for the presence of cardiac cellular hypertrophy, we examined WT and miR-1 TG hearts at P10 by staining for wheat germ agglutinin, which labels the myocardial interstitium and allows direct visualization of cell boundaries. Quantification of myocyte cross sectional area in miR-1 TG animals revealed a significant enlargement in cell size as compared to WT littermates (Supplementary Figures S3A–C). Although a previous study defined abnormalities in sarcomere assembly that contribute to hypertrophy, reduced cell number could also theoretically contribute to abnormal growth by triggering hypertrophy as a compensatory mechanism. To test directly whether miR-1 TG hearts had reduced cell number, we counted the number of cell layers between endocardium and epicardium at several sites along the ventricular walls in miR-1 TG P10 hearts and WT littermates. As expected, miR-TG hearts exhibited a lower cell count as compared to WT littermates, despite an overall increase in wall thickness and cardiac mass (Supplementary Figure 3D).

## Discussion

In this report, we have used a transgenic mouse line with miR-1 overexpression under the control of the Myh6 promoter to define the impact of increased embryonic miR-1 dosage on structure and function of the VCS. Our main findings are (1) the Purkinje system is hypoplastic and exhibits abnormal function in neonatal and adult miR-1 TG mice, and (2) premature upregulation and overexpression of miR-1 in the mid-embryonic and perinatal period inhibits proliferation of PF precursors, possibly through direct regulation of the cyclin-dependent kinase Cdk6. Taken together, our findings corroborate previous reports of arrhythmias caused by increased levels miR-1, and demonstrate that the timing of miR-1 expression is important for development of the VCS.

### Effect of Premature and Increased MiR-1 Expression on Conduction System Proliferation

MiR-1 is the most abundant microRNA in the adult mouse heart, accounting for 35–40% of the total amount of miRNA (Rao et al., 2009) MiR-1 directly targets connexin-43 (Yang et al., 2007), KcnJ2 (Yang et al., 2007), Kcne1 (Jia et al., 2013), Ncx1 (Kumarswamy et al., 2012), PP2A/RyR2 (Terentyev et al., 2009), and Hcn4 (D’Souza et al., 2014) in the adult heart (reviewed in Liao et al., 2016), contributing to the arrhythmias that we and others observed in miR-1 TG adult hearts.

However, in the developmental context, miR-1 is an established negative regulator of cellular proliferation. Accordingly, during heart development, miR-1 levels are relatively low, but rise steeply during the maturation period beginning in late development, supporting the notion that the timing of miR-1 up-regulation is an important determinant of the reduction in proliferation seen during perinatal cardiac maturation (de Boer et al., 2012). Further supporting such a role, loss of miR-1 increases the fraction of cardiomyocytes actively proliferating and prolongs the proliferative period in early perinatal development (Zhao et al., 2007), while overexpression of miR-1 early in embryogenesis results in developmental arrest due to decreased proliferation (Zhao et al., 2005).

The present work is the first to focus on VCS morphogenesis in the context of developmental dysregulation of miR-1 expression. Mammalian PF cells differentiate from a slowly proliferating bipotent pool of ventricular trabecular cardiomyocytes (Gourdie et al., 1995; Miquerol et al., 2010), and subsequently PF cells (located at the tips of trabeculations) proliferate more slowly than compact myocardium (de Boer et al., 2012), resulting in a precise ratio of PF to working myocardium. The gradient in proliferative rate within the developing myocardium is maintained, in part, by the three pocket proteins Rb, p107, and p130, which collectively regulate cell cycling in PF cells and trabecular cardiomyocytes through their interactions with E2F transcription factors. In their complete absence, the PFs and trabecular myocardium exhibit excessive proliferation, leading to compromised cardiac function (Park et al., 2013).

**FIGURE 6:**
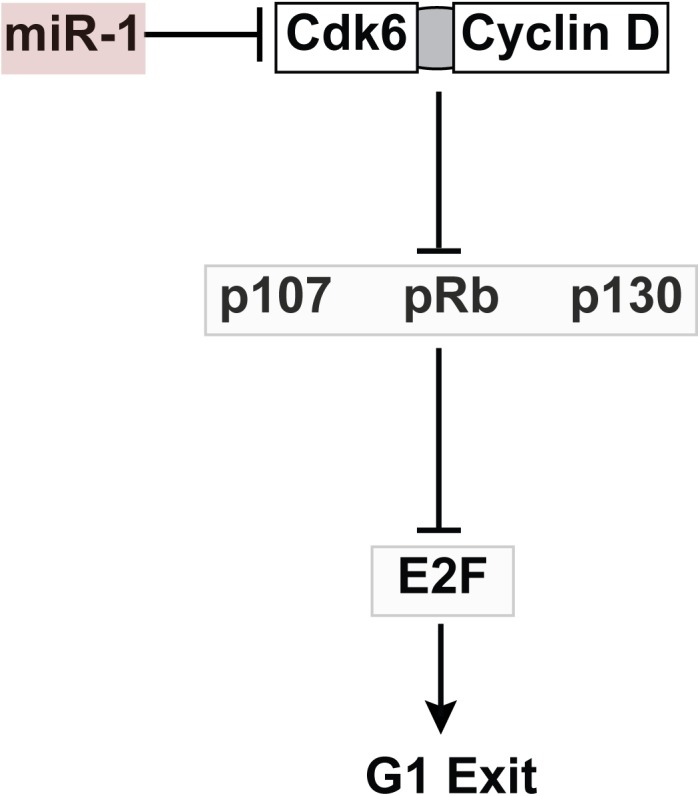
Working Model for Role of MiR-1 in VCS Proliferation. MiR-1 negatively regulates Cdk6, which binds to Cyclin D to inhibit the action of the pocket proteins, negative regulators of VCS cellularity. The net effect of Cdk6 reduction is increased activity of pocket proteins and thus reduced proliferation in the VCS.

Cdk4 and Cdk6 act upstream of the pocket proteins in regulating embryonic cardiomyocyte cell cycling (Ahuja et al., 2007). While Cdk6 is an established cell cycle regulator in embryonic myocardium, a role for Cdk6 specifically in PF development has not been defined. A previous report identified and validated a conserved miR-1 target site in rat Cdk6 leading us to examine this cell cycle regulator in our miR-1 TG model (Yuan et al., 2016). We confirmed that Cdk6 levels are reduced in miR-1 TG hearts, and that mouse Cdk6 is also a miR-1 target. Taken together, these data provide support for our hypothesis that Cdk6 may be an important mediator of the negative effect of miR-1 on embryonic cardiomyocyte proliferation (Figure 6), although we cannot exclude the possibility of other relevant miR-1 target genes that might contribute to the observed phenotype, nor can we exclude the possibility of non-cell autonomous effects of miR-1 on proliferation of VCS precursor cells.

### Relationship of PF Hypoplasia to VCS Function

In addition to observing a negative effect of miR-1 on embryonic trabecular and VCS proliferation, we also note that miR-1 negatively regulates proliferation throughout the myocardium, including the compact zone, leading to a reduction in cell count in the mature heart. Thus, on the surface it may seem puzzling that a global reduction in myocardial proliferation would manifest predominantly via cardiac conduction system dysfunction. One explanation is that conduction tissue may be more sensitive to reduction in total cell number than working myocardium. Careful lineage tracing has demonstrated that by the end of cardiac development and maturation, VCS cells comprise about 1% of the total number of cardiomyocytes. Thus although working cardiomyocytes also have reduced numbers in miR-1 TG animals, they retain the capacity to hypertrophy and can thereby compensate for a decrease in force generating units by increasing the force generated per unit. VCS cells, in contrast, may have limited capacity to change their conduction properties to compensate for even a modest reduction of VCS cell number. This difference between VCS cells and working myocytes could explain why contractile function was relatively preserved in miR-1 TG hearts despite severe disruptions in VCS function. In addition, our finding of reduced Cx-40 expression specifically in VCS cells and globally reduced connexin-43 expression with overall hypertrophy would likely further exacerbate conduction failure by worsening source-sink matching at the PF-myocardium interface. Testing this hypothesis directly with optical mapping of the PF-myocardium junction is beyond the scope of the present work. However, in future work the miR-1 TG adult mouse could serve as an instructive model of how impulse propagation can be compromised in the setting of VCS degeneration and cardiomyopathy.

### Abnormal Cardiac Growth and Early Death in MiR-1 TG Mice

Previous studies that have examined miR-1 overexpression in the adult heart have also noted the presence of progressive AV block, ventricular hypertrophy, and ultimately age-dependent ventricular enlargement with contractile dysfunction. These striking phenotypes have been attributed to upstream effects of miR-1 on a number of specific target genes, including some that regulate impulse propagation and arrhythmogenesis (Zhang et al., 2013), and others that regulate sarcomere assembly (Ai et al., 2012). Here, we demonstrate that in addition to these effects on gene expression in adult hearts, early developmental up-regulation and overexpression of miR-1 has important effects on cardiac morphogenesis and cellularity in the VCS and elsewhere. Although it is not possible to define with certainty the relative contribution of each of these factors to the complex adult phenotype, based on our findings of early-onset prolonged QRS duration in neonatal mice in the absence of any other cardiac phenotypes at that time, we propose that VCS hypoplasia and dysfunction make a significant contribution to abnormal impulse propagation in the postnatal ventricles in miR-1 TG mice, while reduced ventricular cell number could result in hypertrophy as part of an adaptive response.

### Relevance to Human Heart Disease

Although miR-1 dysregulation has been implicated in the pathogenesis of human persistent atrial fibrillation (Girmatsion et al., 2009), a role for miR-1 in regulating AV conduction and VCS function in humans has not yet been defined. However, it is notable that mutations in Nkx2.5, an upstream repressor of miR-1, can cause progressive cardiac conduction disease (PCCD), raising the possibility that this pathway may be relevant to the pathophysiology of at least some forms of PCCD. Further work will be required to test whether miR-1 might play a role in congenital or acquired conduction system disease in humans, and to test whether alteration of miR-1 dosage during defined temporal windows might have a therapeutic role in conduction system disease. In theory, transient reduction of miR-1 dosage in the VCS might offer a means to rescue VCS proliferation in diseases caused by VCS malformation.

## Data Availability

All datasets generated for this study are included in the manuscript and/or the Supplementary Files.

## Author Contributions

VV designed and performed the experiments, analyzed the data, and wrote the manuscript. ES designed and performed the experiments, analyzed the data, and critically edited the manuscript. DS designed the experiments and critically edited the manuscript. GG, YZ, and ME designed and performed the experiments and critically edited the manuscript.

## Conflict of Interest Statement

The authors declare that the research was conducted in the absence of any commercial or financial relationships that could be construed as a potential conflict of interest.

## Supplementary Material

FIGURE S1 Immunohistochemistry of Conduction Tissue in MiR-1 TG Mice. **(A)** Connexin-40 staining of WT (top panels) and TG (bottom panels) demonstrates connexin-40+ subendocardial Purkinje cells. The boxed regions are magnified in the right panels to show connexin-40 plaques in individual VCS cells. **(B)** Quantification of fluorescence intensity of Cx40 plaques relative to background shows a significant reduction in Cx40 protein in TG as compared to WT cells (*n* = 40 WT, *n* = 32 miR-1 TG, “^∗∗∗^” denotes *p* < 0.001). **(C)** Immunohistochemistry for NaV1.5 (left panels) was similar between WT and miR-1 TG, while Cx43 protein expression (right panels) was reduced in miR-1 TG hearts. **(D)** Immunohistochemical staining of AV node with Hcn4 (left) and AV bundle and right bundle branch with Cx40 (right) in WT (top) and miR-1 TG (bottom) adult hearts. **(E)** Acetylcholinesterase staining of WT (top) and miR-1 TG (bottom) AV node (left) and AV bundle (right). Scale bars = 50 microns.

FIGURE S2 MiR-1 TG Mice Exhibit Cardiac Enlargement, AV block, and Early Death. **(A)** Gross examination of miR-1 TG hearts versus WT littermate hearts at 4 time points revealed normal cardiac structure with modest age-dependent enlargement in miR-1 TG hearts. **(B)** Quantification of heart weight to body weight ratio (HW/BW) demonstrated that miR-1 TG animals are similar to WT littermates at birth, but subsequently develop an increase in HW/BW ratio. Abbreviations: P0, postnatal day 0; W, weeks postnatal, NS, non-significant. “^∗∗∗^” denotes *P*-value less than 0.001. **(C)** Examples of spontaneous AV block occurring in three different awake miR-1 TG animals with implanted ECG transmitters. A simultaneous tracing obtained from a WT littermate is shown for comparison (top tracing). P waves that are not followed by QRS complexes, reflecting AV block, are denoted with arrows. AV block was observed in all miR-1 TG mice but in none of the WT littermates. **(D)** Kaplan-Meier curves for miR-1 TG and WT littermates demonstrate early lethality for miR-1 TG animals.

FIGURE S3 Myocardial Hypertrophy and Cellularity in MiR-1 TG Mice. **(A)** Ventricular wall of WT (left) and miR-1 TG (right) hearts at P10 stained for wheat germ agglutinin demonstrates decreased cell number in miR-1 TG hearts. Scale bars = 100 microns. **(B)** Closer view of wheat germ agglutinin staining shows increased cross sectional area of myocardial cells in TG (right) versus WT littermates (left). **(C)** Quantification of cellular cross-sectional area in miR-1 TG versus WT P10 mouse hearts. **(D)** Quantification of number of cell layers between epicardium and endocardium, a surrogate measure of cell number, in left ventricle and right ventricle of WT and miR-1 TG P10 hearts. “^∗∗∗^” denotes *p* < 0.001.

TABLE S1 Echocardiographic Parameters in Awake MiR-1 TG and WT Adult Mice.

TABLE S2 Quantitative PCR Probes.

VIDEO S1 Optical projection tomography was used to visualize the VCS in miR-1 TG; Irx3-LacZ neonatal hearts and Irx3-LacZ littermates after fixing, staining with bluo-gal, clearing, and scanning. Three-dimensional reconstructions of virtual sections demonstrate markedly reduced Purkinje Fibers in the miR-1 TG hearts, similar to findings from crosses to CCS-LacZ.

## Abbreviations

PF: Purkinje fiber
miR-1: microRNA-1
VCS: ventricular conduction system.
