# Supplementary figures and images for: Premature MicroRNA-1 Expression Causes Hypoplasia of the Cardiac Ventricular Conduction System
Source: Front Physiol. 2019 Mar 18;10:235. doi: 10.3389/fphys.2019.00235 (PMC6431665; doi:10.3389/fphys.2019.00235)

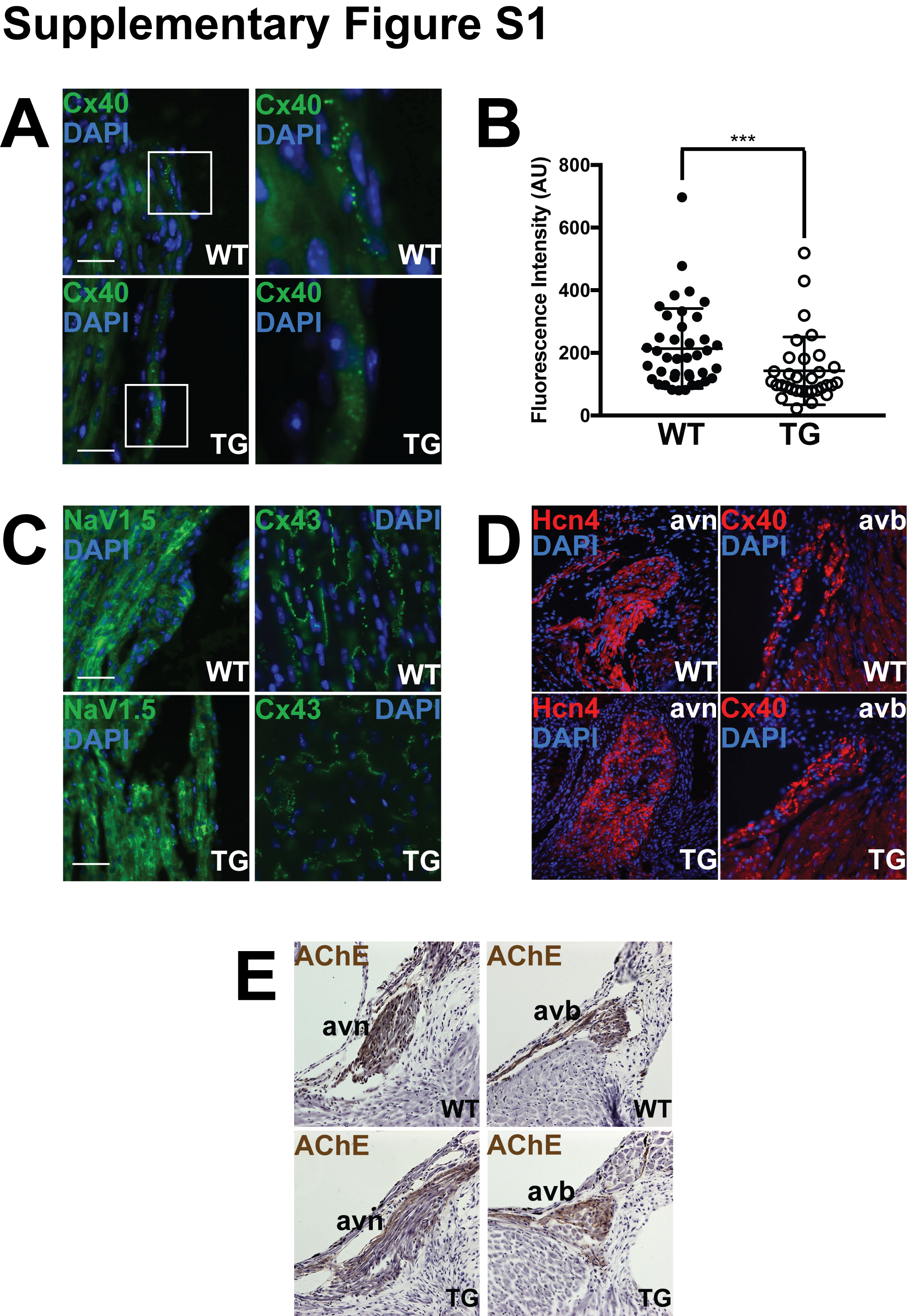

Supplement: FIGURE S1 — Immunohistochemistry of Conduction Tissue in MiR-1 TG Mice. (A) Connexin-40 staining of WT (top panels) and TG (bottom panels) demonstrates connexin-40+ subendocardial Purkinje cells. The boxed regions are magnified in the right panels to show connexin-40 plaques in individual VCS cells. (B) Quantification of fluorescence intensity of Cx40 plaques relative to background shows a significant reduction in Cx40 protein in TG as compared to WT cells (n = 40 WT, n = 32 miR-1 TG, “∗∗∗” denotes p < 0.001). (C) Immunohistochemistry for NaV1.5 (left panels) was similar between WT and miR-1 TG, while Cx43 protein expression (right panels) was reduced in miR-1 TG hearts. (D) Immunohistochemical staining of AV node with Hcn4 (left) and AV bundle and right bundle branch with Cx40 (right) in WT (top) and miR-1 TG (bottom) adult hearts. (E) Acetylcholinesterase staining of WT (top) and miR-1 TG (bottom) AV node (left) and AV bundle (right). Scale bars = 50 microns. [file Image_1.tif]

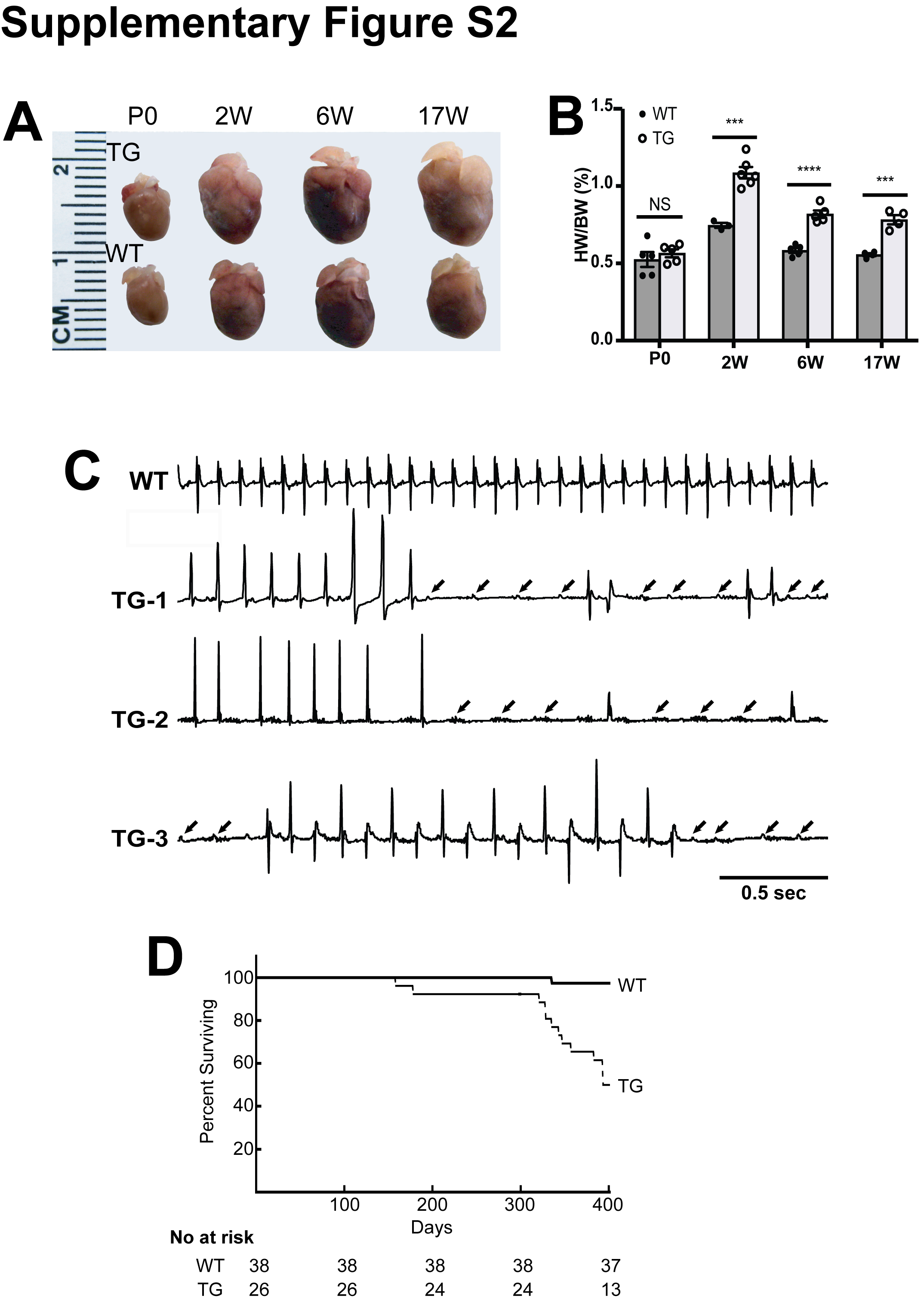

Supplement: FIGURE S2 — MiR-1 TG Mice Exhibit Cardiac Enlargement, AV block, and Early Death. (A) Gross examination of miR-1 TG hearts versus WT littermate hearts at 4 time points revealed normal cardiac structure with modest age-dependent enlargement in miR-1 TG hearts. (B) Quantification of heart weight to body weight ratio (HW/BW) demonstrated that miR-1 TG animals are similar to WT littermates at birth, but subsequently develop an increase in HW/BW ratio. Abbreviations: P0, postnatal day 0; W, weeks postnatal, NS, non-significant. “∗∗∗” denotes P-value less than 0.001. (C) Examples of spontaneous AV block occurring in three different awake miR-1 TG animals with implanted ECG transmitters. A simultaneous tracing obtained from a WT littermate is shown for comparison (top tracing). P waves that are not followed by QRS complexes, reflecting AV block, are denoted with arrows. AV block was observed in all miR-1 TG mice but in none of the WT littermates. (D) Kaplan-Meier curves for miR-1 TG and WT littermates demonstrate early lethality for miR-1 TG animals. [file Image_2.tif]

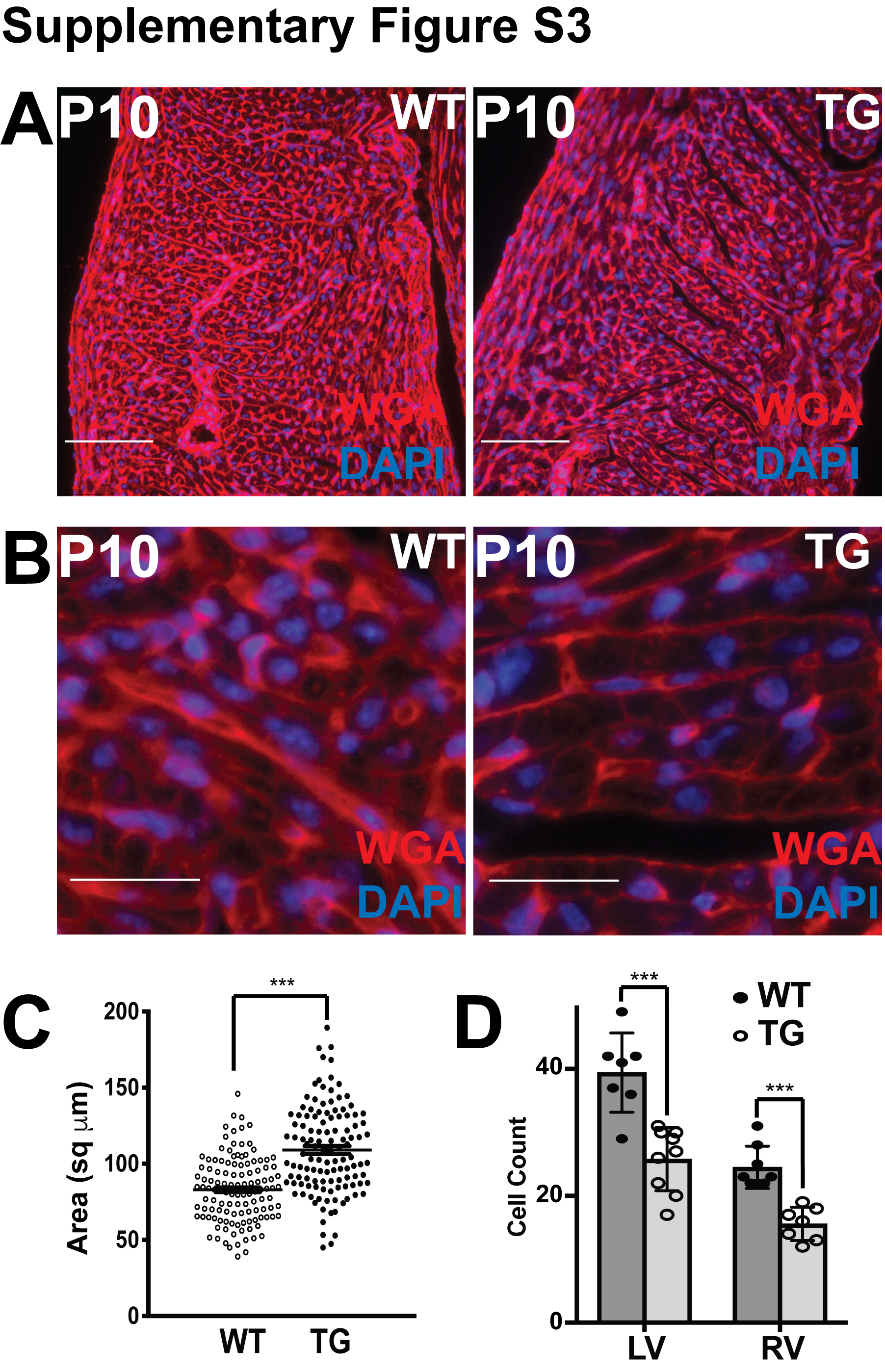

Supplement: FIGURE S3 — Myocardial Hypertrophy and Cellularity in MiR-1 TG Mice. (A) Ventricular wall of WT (left) and miR-1 TG (right) hearts at P10 stained for wheat germ agglutinin demonstrates decreased cell number in miR-1 TG hearts. Scale bars = 100 microns. (B) Closer view of wheat germ agglutinin staining shows increased cross sectional area of myocardial cells in TG (right) versus WT littermates (left). (C) Quantification of cellular cross-sectional area in miR-1 TG versus WT P10 mouse hearts. (D) Quantification of number of cell layers between epicardium and endocardium, a surrogate measure of cell number, in left ventricle and right ventricle of WT and miR-1 TG P10 hearts. “∗∗∗” denotes p < 0.001. [file Image_3.tif]
