# Supplementary material for: Premature MicroRNA-1 Expression Causes Hypoplasia of the Cardiac Ventricular Conduction System
Source: Front Physiol. 2019 Mar 18;10:235. doi: 10.3389/fphys.2019.00235 (PMC6431665; doi:10.3389/fphys.2019.00235)
Supplement: TABLE S1 — Echocardiographic Parameters in Awake MiR-1 TG and WT Adult Mice. [file Table_1.docx]

**Table S1: Quantitative PCR Probes**

**Taqman mRNA qPCR Assays**

| **Gene** | **Taqman Assay** | **Reference Sequence** | **Base Location** | **Exon Boundary** | **Amplicon Length (bp)** |
| --- | --- | --- | --- | --- | --- |
| Cdk6 | Mm00438163 | NM_009873.2 | 595 | 3-4 | 87 |
| Gapdh | Mm99999915 | NM_001289726.1 | 117 | 2-3 | 107 |
| Gata4 | Mm00484689 | NM_008092.3 | 1392 | 3-4 | 84 |
| Gja5 | Mm00433619 | NM_001271628.1 | 732 | 3-3 | 84 |
| Hcn4 | Mm01176086 | NM_001081192.1 | 1367 | 3-4 | 70 |
| Irx3 | Mm00500463 | NM_001253822.1 | 1896 | 3-4 | 113 |
| Nkx2-5 | Mm01309813 | NM_008700.2 | 545 | 1-2 | 56 |
| Tbx5 | Mm00803521 | NM_011537.3 | 1632 | 8-9 | 91 |

**Taqman MicroRNA qPCR Assays**

| **MiRNA** | **Taqman Assay** | **Reference Sequence (miRBase)** | **Mature miRNA Sequence** |
| --- | --- | --- | --- |
| miR-1 | 000385 | MIMAT0000123 | UGGAAUGUAAAGAAGUAUGUA |
| miR-133 | 000458 | MIMAT0000145 | UUGGUCCCCUUCAACCAGCUGU |
| miR-143 | 002249 | MIMAT0000247 | UGAGAUGAAGCACUGUAGCUC |
| miR-16 | 000391 | MIMAT0000527 | UAGCAGCACGUAAAUAUUGGCG |
| miR-208 | 000511 | MIMAT0000520 | AUAAGACGAGCAAAAAGCUUGU |
| miR-24 | 000402 | MIMAT0000219 | UGGCUCAGUUCAGCAGGAACAG |
| miR-499 | 001352 | MIMAT0003482 | UUAAGACUUGCAGUGAUGUUU |
| U6 | 001973 | NR_004394 (NCBI) | N.A. |
