# Supplementary material for: Premature MicroRNA-1 Expression Causes Hypoplasia of the Cardiac Ventricular Conduction System
Source: Front Physiol. 2019 Mar 18;10:235. doi: 10.3389/fphys.2019.00235 (PMC6431665; doi:10.3389/fphys.2019.00235)
Supplement: TABLE S2 — Quantitative PCR Probes. [file Table_2.docx]

**Table S2. Echocardiographic Parameters in Awake MiR-1 TG and WT Adult Mice**

| Parameter | miR-1 TG (*n* = 9) | WT (*n* = 6) | *p* |
| --- | --- | --- | --- |
| Septum (diastole) (mm) | 0.93 ± 0.02 | 0.90 ± 0.08 | 0.66 |
| LV diameter (diastole) (mm) | 3.9 ± 0.2 | 3.4 ± 0.2 | 0.05 |
| Posterior Wall (diastole) (mm) | 0.91 ± 0.05 | 0.91 ± 0.05 | 0.98 |
| Septum (systole) (mm) | 1.63 ± 0.05 | 1.54 ± 0.06 | 0.29 |
| LV diameter (systole) (mm) | 2.0 ± 0.2 | 1.7 ± 0.2 | 0.29 |
| LV Posterior Wall (systole) (mm) | 1.54 ± 0.08 | 1.47 ± 0.05 | 0.51 |
| Fractional Shortening (%) | 50 ± 3 | 51 ± 4 | 0.77 |
